# Prevalence and risk factors for inter-generational Sex: a cross-sectional cluster survey of Barbadian females aged 15–19

**DOI:** 10.1186/1472-6874-13-53

**Published:** 2013-12-27

**Authors:** Nicole Drakes, Clarissa Perks, Alok Kumar, Kim Quimby, Colin Clarke, Rajul Patel, Ian R Hambleton, R Clive Landis

**Affiliations:** 1 National HIV AIDS Commission, National AIDS Program, Bridgetown, Barbados; 2 Department of Genito Urinary Medicine & HIV, Southampton University, Southampton, UK; 3 Department of Pediatrics, Faculty of Medical Sciences, University of the West Indies, Bridgetown, Barbados; 4 Chronic Disease Research Centre, The University of the West Indies, Bridgetown, Barbados; 5 Department of Youth Affairs, Ministry of Youth, Family & Sports, Bridgetown, Barbados; 6 Edmund Cohen Laboratory for Vascular Research, Chronic Disease Research Centre, University of the West Indies, Bridgetown BB11115, Barbados

## Abstract

**Background:**

The aim of this study was to establish the prevalence and risk factors for intergenerational (IG)-sex in females aged 15–19 residing in Barbados.

**Methods:**

This cross sectional cluster survey was conducted in a 2.6% national sample in the age range (n = 261) recruited from multiple polling districts chosen with a probability proportional to size. Consent was obtained from participants aged ≥18 years or from parents/guardians of participants <18 years, with participant assent. The prevalence of age at first sex was analyzed using a life table approach and risk factors for IG sex (defined as sexual relations with a male 10 or more years older) were analyzed by logistic regression, adjusting for age.

**Results:**

51.0% of adolescent females in the survey reported ever having had sex, among whom prevalence of IG-sex was 13.2% (95% CI: 6.7-19.8) at first sex, 29.0% (22.3-35.7) within the preceding twelve months, and 34.8% (24.3-45.4) ever. Condom use at first sex was positively related to willingness to have sex (F statistic = 9.8, p = 0.001). The strongest determinant for IG-sex was age of sexual debut (age adjusted Odds Ratio [95% CI]: 0.58[0.41-0.83]), followed by money or gifts received from the partner (2.91[1.17-7.23] and self-esteem (0.33[0.11-0.95]).

**Conclusions:**

The survey establishes a high rate of IG-sex in Barbados, a ‘high income’ country. Most insightful is that risk of IG-sex nearly halved for every year at which first sex was delayed. A high proportion of coerced sex was reported at first sexual experience and this was linked to poor condom use. Affirmative prevention approaches are recommended to boost self-acclamation of adolescent women within less coercive relationships, especially during their first sexual encounter.

## Background

The Caribbean region has the second highest prevalence of HIV worldwide, behind sub-Saharan Africa, and is one of two regions (the other being Africa) where the proportion of young women living with HIV in the 15–24 age group outnumbers males [1,2]. In Barbados the overall prevalence of HIV remains higher in men, but in the age group 10–19 it is more prevalent in women [2]. This signifies a major shift for a disease originally concentrated within the male population. Multiple, intergenerational and transactional sexual partnering, along with cultural norms and gender roles that limit women’s sexual health choices, are implicated as driving this epidemiologic transition to young women [3-5].

Inter-generational (IG)-sex is generally defined in the literature as heterosexual intercourse with a non-marital partner ten or more years older [6-8]. Also referred to as cross generational sex or the ‘sugar daddy’ syndrome, it is primarily characterised by relationships between younger women and older men [9,10]. Research in Africa supports the existence of a link between IG-sex and HIV infection among adolescent females. In sub-Saharan Africa, adolescent women aged 15–24 were between 2 to 4 times more likely to be infected than their male contemporaries [11], while research by Kelly et al. (2003) identified large age differences in sexual relationships as a contributing factor to HIV prevalence [12]. A study of adolescent women aged 15–24 in rural Zimbabwe concluded that each one-year increase in age difference between partners was associated with a 4 percent increase in risk of HIV infection [13].

Transactional sex, often defined as sex in exchange for financial or material gain, can place women’s health at risk [7,12-15]. These relationships, commonly intergenerational, are linked with reduced condom use [4,6]. The greater the asymmetry in partner age and wealth, the less room for sexual negotiation on the female’s part [9,16-19]. Despite high levels of HIV awareness and education in the Caribbean, levels of reported condom use remain inconsistent [5,20-23]. As in other parts of the world, the ability to exercise condom use by young females is impacted by entrenched gender stereotypes, cultural attitudes towards sex, asymmetric power distribution within relationships, sexual violence, and unequal access to resources [4,17,24-27].

Information on IG-sex is quite sparse for the Caribbean. However, Demographic Health Surveys and KABP studies have estimated IG-sex prevalence among sexually active females aged 15–19 years within the preceding 12 months ranging from 3.7% in St. Kitts & Nevis, 6% in The Dominican Republic, 7.6% in Dominica, 9.8% in Haiti, 17.4% in Antigua & Barbuda, and 17.7% in Grenada [20,28]. Two focus group studies conducted in Jamaica indicated that girls having relationships with older men were regarded as ‘common’ and ‘normal’ among peers, though not necessarily by the family [29,30]. A series of Demographic Health Surveys in African countries revealed a range of IG-sex prevalence in 15–19 year olds from 3.3% in Malawi, 9.6% in Ghana, 13.7% in Tanzania, 19.3% in Uganda, and 25.5% in Nigeria [31-35]. The transactional element that often characterizes these types of relationship is a strong motivating factor, not only through accruing material or social benefits but also by affirmation of self-worth [7,36-38]. Indeed, the meaning of transactional sex within a given cultural context remains the subject of debate [38,39].

Our survey therefore sought to measure prevalence of IG-sex as a primary outcome measure in a nationally representative survey, conducted in adolescent females aged 15–19 residing in Barbados. Determinants examined for IG-sex included transactional sex, age, age at first sex, religious worship, and psycho-social measures of self-esteem, sense of personal power, and peer pressure. The findings were contrasted with the determinants for sex with any age partner in the same survey population.

## Methods

### Study population

The study was completed among adolescent females aged 15 to 19 years, who were sampled from multiple polling districts (chosen with a probability proportional to size) to achieve a target 3.0% sample of the population of 15–19 year old women in Barbados (there were 10,045 women in this age range according to the adjusted 2000 census). The survey returned 261 completed questionnaires, an 87% response rate that yielded a final 2.6% sample of the population in the age group. The sample design was a two-stage random sample of girls aged between 15 and 19. Stage 1 consisted of selecting polling districts with probability proportional to size. The island is divided into 313 polling districts and a 5% sample of districts yielded 16 districts. The second stage of sampling was done by interviewers within each polling district, who selected every fifth household for inclusion after a randomly chosen start. All eligible girls within each household were selected for inclusion in the survey.

### Informed consent

All surveys were conducted with informed consent and IRB approval, in accordance with the principles of the Declaration of Helsinki as revised in 2000. The survey was interviewer-administered with Youth Commissioners of the Youth Department selected on the basis of experience in fieldwork and working with young people. A number of provisions were taken to maintain confidentiality in this sensitive youth survey: interviewers were assigned to a polling district outside of the area in which they generally worked; deliberate non-recording of personal identifiers such as names and addresses ensured anonymity of respondents and confidentiality of the data collected; written informed consent was obtained from participants aged 18 years or older, or from parents/guardians of participants aged 17 years or younger, with the assent of the participant; parents or guardians were not permitted to sit-in on interviews. Interviewers were trained to answer any queries posed by the respondents or their parents/guardians. Ethical approval for the study protocol was obtained from the ethics committee serving the Barbados Ministry of Health and The University of the West Indies, as well as the IRB of the University of Southampton, UK.

### Survey instrument

The survey instrument was designed by The University of the West Indies (UWI) and The National HIV/AIDS Commission (NHAC) based on the Interpersonal Violence (IPV) survey and previous KABP surveys conducted in Barbados, Jamaica and several African states [22,23,26]. The questionnaire consisted of 98 items, all closed-ended questions, seeking to capture socio-demographic data on the target group as well as information on the respondent’s sexual history, economic indicators, the practice of transactional sex, relationship dynamics (same-age vs intergenerational partners), and possible determinants of sex (sense of self-esteem, personal power, peer pressure, perceived financial status, and religious worship attendance). Validated instruments included scales for peer pressure, personal power, and self-esteem [40-43]. “Pregnancy/STD Prevention” of the Sexual Assertiveness Scale (Morokoff) was included, but not subscales on “Initiation” and “Refusal” that were considered too sensitive for this youth survey [44]. The experience at first sex, including condom use, willingness to have sex, and partner’s age, was assessed using questions asked in successive KABP surveys in Barbados [22]. Religious worship was assessed with the question: “do you attend religious service?”, scored on a scale “never, a few times a year, monthly, weekly, more than weekly”. Perceived financial distress in this youth population was assessed by the following subjective statements: “I have enough money to cover my daily necessities” and “I have enough money to cover luxuries”, scored on a scale “strongly agree, agree, disagree, strongly disagree”. A pre-test of the survey instrument was conducted on a similar population to the respondent population with the aim of identifying any weaknesses in questionnaire design and providing practice for fieldworkers. Double data entry was performed to minimize transcription error.

### Key outcome measures

The primary outcome measure was inter-generational sex. The UNAIDS definition of inter-generational relationships (also referred to as cross-generational) was used to guide the survey [8]. IG Sex was therefore defined as heterosexual intercourse with an unmarried male partner 10 or more years older. IG prevalence was measured using 3 items: respondent self-reports of sexual intercourse with a male partner 10 or more years older at the first sexual encounter; sexual intercourse within the past 12 months with a male partner 10 or more years older; and ever having had sexual intercourse with a male partner 10 or more years older. Transactional sex is the exchange of money, food and other items for sex [10,14,15,38]. The survey asked respondents if they had ever exchanged sex in exchange for money or gifts, and the question was repeated for same-age and older partners. Several predictors of IG sex were incorporated into the survey, some of which were based on individual items: age at first sexual encounter; age of first sexual partner; religious service attendance; and psychosocial variables of self-esteem, sense of personal power and peer pressure.

### Statistical methods

The prevalence rates for inter-generational sex (yes/no) were calculated with associated 95% confidence intervals for the following scenarios: at first sex, in the past 12-months, and ever. In secondary analyses, associations between selected sexual and psychosocial characteristics and the probability of reporting having had sex or inter-generational sex were examined used a series of logistic regression analyses, reporting odds ratios (unadjusted and age-adjusted) and their associated 95% confidence intervals. For all prevalence calculations and regression analyses, standard errors and 95% confidence intervals reflect the two-stage survey design and the increased correlation of participants recruited from a single household. Such survey designs inflate the standard errors of any point estimates, and a Taylor linearization was used for all variance estimation. The change in the proportion of respondents reporting sexual activity with age was quantified using a univariate logistic regression, and reported using an odds ratio and associated 95% confidence interval. To calculate the age at first sex a life table approach was used, which calculated the cumulative proportion having sex at each year of age up to 19. The technique allowed for those adolescents who had never had sex to be censored at their current age. Although all analyses relied on 95% confidence intervals to describe the strength of associations between possible predictors and model outcomes, for completeness p-values were occasionally calculated for primary associations of interest, and in these situations exact p-values were presented to clarify the strength of the statistical relationships. All analyses were performed using Stata statistical software (version 13, StataCorp LP, College Station, Texas, USA).

## Results

### Characteristics of survey population stratified by sexual history

The survey returned 261 completed questionnaires, representing an 87% response rate and an overall 2.6% sample of the female population within the 15–19 year age range in Barbados. The average item non-response rate was minimal (5.3%). The sexual history of 259 females who responded to this item is shown in Table 1. Respondents were stratified according to age and type of sex engaged in (i.e. never had sex, had sex but not IG-sex, had IG-sex). 127 respondents reported never having had sex, 86 respondents reported having had sex but not IG-sex, while 46 respondents reported having had IG-sex. The proportion of adolescents who reported having had sex was strongly age-related, with prevalence of any sex (oral, vaginal, anal) rising from 30% among 15 year-olds to 84% among 19 year-olds (*data not shown*; F statistic = 7.4, p < 0.001). Table 1 also categorizes responses to questions on perceived financial distress and religious worship attendance, stratified according to sexual history. This group of adolescent females reported perceived financial distress, with almost 40% of the total survey population disagreeing or strongly disagreeing that they had sufficient money to cover daily necessities, while over two thirds felt they did not have enough money to cover luxuries. Religious worship attendance revealed 40.9% of respondents attended service regularly (weekly or > weekly), 20.6% attended service occasionally, and 38.4% never attended a religious service. Respondents were Christian (54.9%), non-Christian (6.2%), and of no religious affiliation (38.9%).

**Table 1:** Selected characteristics of 259 Barbadian females aged 15 to 19, stratified by sexual history (never had sex, had sex but not inter-generational sex, had inter-generational sex)

	**Never had sex N = 127**	**Had sex but not inter-generational sex N = 86**	**Had inter-generational sex N = 46**	**Total N = 256**
**Characteristic**	**n (percent)**	**n (percent)**	**n (percent)**	**n (percent)**
**Age (n = 256)**				
15	39 (30.7)	9 (10.8)	8 (17.4)	56 (21.9)
16	38 (29.9)	9 (10.8)	3 (6.5)	50 (19.5)
17	23 (18.1)	27 (32.5)	5 (10.9)	55 (21.5)
18	20 (15.8)	18 (21.7)	12 (26.1)	50 (19.5)
19	7 (5.5)	20 (24.1)	18 (39.1)	45 (17.6)
**Enough money to cover necessities (n = 251)**				
Strongly agree	12 (9.8)	6 (7.3)	0 (-)	18 (7.2)
Agree	60 (48.8)	47 (57.3)	27 (58.7)	134 (53.4)
Disagree	39 (31.7)	23 (28.1)	17 (37.0)	79 (31.5)
Strongly disagree	12 (9.8)	6 (7.3)	2 (4.3)	20 (8.0)
**Enough money to cover luxuries (n = 245)**				
Strongly agree	7 (5.8)	1 (1.2)	1 (2.3)	9 (3.7)
Agree	35 (29.2)	22 (27.2)	13 (29.6)	70 (28.6)
Disagree	61 (50.8)	48 (59.3)	27 (61.4)	136 (55.5)
Strongly disagree	17 (14.2)	10 (12.4)	3 (6.8)	30 (12.2)
**Do you attend religious services (n = 240)**				
Never	31 (25.8)	39 (47.6)	22 (57.9)	92 (38.3)
Less than monthly	6 (5.0)	12 (14.6)	6 (15.8)	24 (10.0)
Monthly	11 (9.2)	11 (13.4)	3 (7.9)	25 (10.4)
Weekly	41 (34.2)	14 (17.1)	4 (10.5)	59 (24.6)
More than weekly	31 (25.8)	6 (7.3)	5 (7.9)	40 (16.7)

### Age at first sex

**Figure 1:**
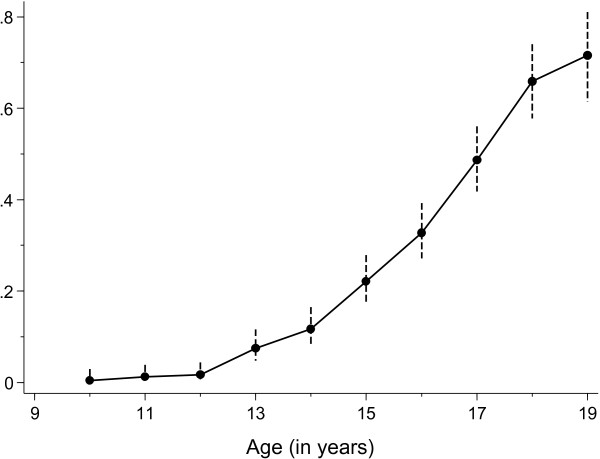
**Age at first sex among 261 Barbadian females aged 15–19.** The distribution of age at first sex was generated from censored observations by survival analysis. The input data were age of the respondent, whether or not they ever had sex and if applicable recalled age at first sex. In calculating a survival function describing the probability of remaining a virgin, reported age at first sex is the ‘death’ event and those who never had intercourse are censored at their current age.

The age at first sex is illustrated in Figure 1. In this group of adolescent females aged 15 to 19 at the time of interview, 7.3% reported their age at first sex before 13 years of age, 11.3% before 14 years, 21.5% before 15 years, 31.8% before 16 years, 46.9% before 17 years, 62.6% before 18 years, and 66.5% before 19 years. Using this censored life table analysis, 25% of participants had had sex by 15 years of age (lower quartile), and 50% had had sex by 17 years of age (median).

### Sexual history and inter-generational sex

The key outcome measure of this survey was the prevalence of IG-sex, reported in Table 2. Overall, 51.0% (95% CI: 42.5 – 59.4, n = 134) of respondents reported ever having had sex and 17.8% (11.3 – 24.3, n = 46) reported a sexual history with a partner 10 or more years older. As a proportion of females who had ever had sex, IG-sex was reported at the first sexual encounter by 13.2% (95% CI: 6.7 – 19.8, n = 16), 29.0% (95% CI: 22.3 – 35.7, n = 31) within the preceding twelve months, and 34.8% (95% CI: 24.3 – 45.4, n = 46) ever. The age difference of the male partner at the first sexual encounter was examined in further detail: the age difference was reported to be 10 or more years older in 13.2% (95% CI: 6.7 – 19.8, n = 16) of respondents, 5–9 years older in 25.6% of respondents (95% CI: 15.6 – 35.7, n = 31), “older, unsure how much” in 14.9% (95% CI: 7.0 – 22.8, n = 18), and of similar age in 46.3% of respondents (95% CI: 37.8 – 54.7, n = 56). Zero percent of girls in the survey reported a younger partner at first sex. Money or gifts were equally likely to be received from a same-age versus an IG partner. Condoms were used by 68/125 of girls during their first sexual encounter (54.4%, CI: 40.4% to 68.4%). Condom use was strongly related to willingness to have sex. A sizeable proportion of girls were either “persuaded to have sex” (30.8%, CI: 21.2% to 40.3%, n = 40) or “did not want to have sex” and (6.9%, CI: 2.9% to 11.0%, n = 9). Condom use was 68% among those who “wanted to have sex” (n = 81), 37% among those who were “persuaded to have sex”, and a condom was never used among those who “did not want to have sex” (F statistic = 9.8, p = 0.001). Multiple partnering in sexually active females showed that 30.2% of respondents reported more than one partner in the past 12 months: 18.9% reported 2 partners, 5.0% 3 partners, 3.3% 4 partners, 3.0% 5 or more partners.

**Table 2:** Sexual history among 261 Barbadian females aged 15 to 19, and selected sexual characteristics among 134 Barbadian females who have had sex at least once

**Characteristics**	**Response**	**Number**	**%**	**95% CI**
**Sexual history: prevalence of sexual activity †**				
Ever had sex?	259 / 261	132	51.0	42.5 to 59.4
Ever had oral sex?	243 / 261	67	27.6	18.7 to 36.4
Ever had vaginal sex?	258 / 261	126	48.8	40.9 to 56.8
Ever had anal sex?	237 / 261	15	6.3	2.7 to 9.9
**Sexual history: prevalence of Inter-Generational sex**				
At first sex	248 / 261	16	6.5	3.1 to 9.8
Within past 12 months	234 / 261	31	13.2	8.3 to 18.2
At any time	259 / 261	46	17.8	11.3 to 24.3
**Characteristics among females who have had sex at least once**				
Inter-Generational sex at first sex	121 / 132	16	13.2	6.7 to 19.8
Inter-Generational sex in past 12 months	107 / 132	31	29.0	22.3 to 35.7
Inter-Generational sex ever?	132 / 132	46	34.8	24.3 to 45.4
Age of partner at first sex	121 / 132			
10 or more years older		16	13.2	6.7 to 19.8
5 to 9 years older		31	25.6	15.6 to 35.7
Older, unsure how much		18	14.9	7.0 to 22.8
About the same age		56	46.3	37.8 to 54.7
Money or gifts from sexual partner?	125 / 132	69	55.2	43.5 to 66.9
Money or gifts from same-age partner?	61 / 69	22	36.1	24.2 to 49.4
Money or gifts from older partner?	61 / 69	20	32.8	21.3 to 46.0
Condom used at first sexual encounter?	125 / 132	68	54.4	40.4 to 68.4
Willingness at first sex?	130 / 132			
wanted to have sex		81	62.3	52.6 to 72.0
persuaded to have sex		40	30.8	21.2 to 40.3
did not want to have sex		9	6.9	2.9 to 11.0
Sex in past 12 months?	129 / 132	105	81.4	70.7 to 92.1
Number of partners in past 12 months?	132 / 132			
0		33	25.0	13.7 to 36.3
1		59	44.7	34.8 to 54.5
2		25	18.9	10.8 to 27.1
3-4		11	8.3	2.7 to 14.0
5+		4	3.0	0.3 to 5.7

† Ever had sex (N = 259 respondents). Compared to Table 1 (N = 256). This was due to three participants without known age.

### Determinants for the likelihood of having had sex (any-age partner)

A range of demographic, economic, and psycho-social factors were examined for their relationship with the likelihood of having had sex (any-age partner) in this adolescent female population. As expected, age was a strong predictor of having had sex, with the odds increasing by 1.87 (95% CI 1.43 – 2.45) for every additional year of age. All other factors were therefore pre-adjusted for age and are presented on the right of Table 3. We could not demonstrate a link between financial security and the likelihood of having had sex, despite the evidence for perceived financial distress captured in Table 1. Religious service attendance was strongly related to having had sex, with those adolescent females not attending church reporting an almost three times greater chance of having had sex (odds ratio 2.86, 95% CI 1.85 – 4.41). More specifically, those either not attending church or attending church monthly or less were more likely to report having had sex compared to those attending church weekly or more than weekly (p = 0.01 or stronger in all cases). Finally, a constellation of psycho-social factors, including self-esteem, sense of personal power, and peer-pressure, were examined for their association with having had sex in this adolescent female population. Whereas increasing peer pressure significantly increased the chance of reporting sexual activity (age adjusted Odds Ratio [95% CI]: 4.4 [2.34-8.29], higher levels of self-esteem (0.10 [0.03-0.37]) and ‘sense of personal power’ (0.42 [0.20-0.85]) statistically significantly decreased the chance. Given the statistically significant effects of peer pressure and religious service attendance on the probability of having had sex, the relationship between peer pressure and religious service attendance was examined as a post-hoc analysis. A regression output demonstrated that the peer pressure index (and so peer pressure) increased among those who did not attend a religious service, relative to those who did (coefficient = 0.26, 95% CI 0.13 to 0.39). Furthermore, the frequency of religious service attendance was associated with a systematic downward trend in peer pressure (p = 0.01).

**Table 3:** Unadjusted and age-adjusted associations for ever having had sex (yes/no)

**Factor**	**N**	**Unadjusted odds ratio (95% CI)**	**Age adjusted odds ratio (95% CI) †**
Age	256	1.87 (1.43 – 2.45)	-
Enough money to cover necessities	250	0.98 (0.64 – 1.49)	1.01 (0.64 – 1.60)
Enough money to cover luxuries	244	1.16 (0.70 – 1.92)	1.40 (0.76 – 2.57)
Religious service attendance (yes/no)	239	2.89 (1.82 – 4.60)	2.86 (1.85 – 4.41)
Frequency of religious service attendance ‡	239		
Never		6.78 (3.16 – 14.54)	7.91 (3.59 – 17.41)
Less than monthly		10.33 (2.48 – 43.01)	7.89 (1.96 – 31.81)
Monthly		4.07 (1.48 – 11.17)	7.28 (1.88 – 28.12)
Weekly		1.51 (0.61 – 3.72)	1.96 (0.76 – 5.04)
More than weekly	-	-	-
**Psychosocial factors**			
Self-esteem	254	0.17 (0.06 – 0.44)	0.10 (0.03 – 0.37)
Sense of personal power	237	0.55 (0.27 – 1.14)	0.42 (0.20 – 0.85)
Peer-pressure	248	2.83 (1.65 – 4.86)	4.40 (2.34 – 8.29)

† Age adjusted logistic regressions, adjusted for effects of current age.

‡ Odds ratios relative to attending religious service “More than weekly”.

### Determinants for inter-generational sex

Unadjusted and age-adjusted odds of reporting IG-sex are shown in Table 4. The unadjusted odds ratio of reporting IG-sex rose by 1.56 for every additional year of age (95% CI 0.91 – 2.68), as expected. More insightful was that the chance of IG-sex decreased as age at first sex increased (age-adjusted odds ratio 0.58, 95% CI 0.41 – 0.83). This amounts to almost a halving of risk for every additional year of age at which the first sexual encounter occurred. There was no association of IG-sex with a perception towards insufficient money to cover necessities (p = 0.97) but there was a mild trend towards perceived financial insufficiency as it related to purchase of luxuries (p = 0.27). Money or gifts from the partner was statistically significantly associated with IG-sex (OR = 2.91, 95% CI 1.17 – 7.23). Religious service attendance was not associated with the odds of reporting IG-sex (p = 0.82). Of the psychosocial factors, we could not demonstrate a link between peer-pressure or sense of personal power with IG-sex. However, self-esteem was statistically significantly associated with IG-sex: i.e. higher self-esteem was associated with a three-fold reduction in IG-sex (OR = 0.33, 95% CI 0.11 – 0.95). Having more than one partner in the past 12 months was associated with IG-sex (age adjusted odds ratio 3.27, 95% CI 1.55 to 6.91, not shown).

**Table 4:** Unadjusted and age-adjusted associations for inter-generational sex (yes/no) in females who had ever had sex

**Factor**	**N**	**Unadjusted odds ratio (95% CI)**	**Age adjusted odds ratio (95% CI) †**
Age	129	1.16 (0.77 – 1.73)	1.56 (0.91 – 2.68)
Age at first sex	120	0.67 (0.50 – 0.90)	0.58 (0.41 – 0.83)
Condom use at first sex	123	1.58 (0.51 – 4.91)	0.66 (0.16 – 2.77)
Willingness at first sex	128	0.86 (0.45 – 1.62)	0.55 (0.26 – 1.78)
Enough money to cover necessities	127	1.21 (0.58 – 2.51)	0.99 (0.46 – 2.11)
Enough money to cover luxuries	124	0.72 (0.35 – 1.51)	0.60 (0.23 – 1.56)
Money or gifts from partner	123	3.35 (1.51 – 7.45)	2.91 (1.17 – 7.23)
Religious service attendance (yes/no)	119	1.48 (0.50 – 4.37)	0.88 (0.27 – 2.87)
Frequency of religious service attendance ‡	119		
Never		1.13 (0.22 – 5.90)	0.54 (0.08 – 3.54)
Less than monthly		1.00 (0.18 – 5.58)	0.72 (0.08 – 6.46)
Monthly		0.60 (0.07 – 5.43)	0.64 (0.07 – 6.22)
Weekly		0.57 (0.18 – 1.77)	0.44 (0.12 – 1.66)
More than weekly	-	-	-
Psychosocial factors			
Self-esteem	127	0.32 (0.16 – 0.64)	0.33 (0.11 – 0.95)
Sense of personal power	122	0.77 (0.33 – 1.83)	1.10 (0.47 – 2.56)
Peer-pressure	128	1.42 (0.71 – 2.86)	1.42 (0.61 – 3.29)
Morokoff subscale (pregnancy/STD risk)	104	1.55 (0.84 – 2.86)	1.37 (0.58 – 3.22)

† Age adjusted logistic regressions, adjusted for effects of current age.

‡ Odds ratios relative to attending religious service “More than weekly”.

## Discussion

The present study is the first to report prevalence rates of IG-sex among adolescent females in Barbados in a nationally representative survey. The rate of IG-sex among sexually active females aged 15–19 is 29.0% (95% CI: 22.3 – 35.7), higher than the only existing survey data from the Eastern Caribbean and comparable to rates at the uppermost range in Africa [20,28,31-35]. The proportion of females who reported IG-sex at their first sexual experience, 13.2% (95% CI: 6.7 – 19.8), was consistent with a KABP survey conducted in Barbados in 2011 that reported 11.4% (unpublished). The relatively high rates of IG-sex reported here are therefore consistent with the claim made in the UNAIDS Caribbean Report of 2008 that it is ‘common practise’ among adolescent girls in the Caribbean to maintain relationships with older men [3].

The high rates of IG-sex were not expected based purely on an assumed link with poverty or lack of education among adolescent girls. In rural Africa, it has been established that poverty, in the context of poor access to education, healthcare, and employment opportunities, urges women into such relationships [7,17,45,46]. Barbados is classified as a ‘high income’ country by the World Bank with a gini coefficient (a measure of income inequality) of 0.39 that places it between the the US (0.41) and the UK (0.36). Barbados also has one of the lowest rates of poverty in the Caribbean and Latin America, and has a high literacy rate according to the United Nations Development Program [47,48]. Despite these favorable indicators and a high degree of knowledge of condom use identified in successive KABP surveys, the present survey uncovered evidence of relationship imbalances with age-disparate partnering particularly at sexual debut.

The first sexual experience was associated with forcible sex or pressure to have sex in nearly 40% of respondents, a worrying finding that is consistent with a pattern of sexual coercion, sexual abuse and high rates of interpersonal violence reported in the Caribbean literature [5,19,26,49-53]. Condom use was strikingly related to willingness to have sex, with a distribution of 2:1:0 in girls who were either willing:persuaded:forced to have sex at their first sexual encounter.

The strongest predictor of IG-sex was the age at first sex. The median age at first sex was 17 years. On the face of it this is later than reported by other studies in the region but apparent discrepancies may be explained by the way age at first sex was reported and by gender differences [50,54,55]. The authoritative multi-country Caribbean Youth Health Survey (CYHS) survey is often quoted out of context [50]. Commentators have reported the headline finding that nearly half of adolescents were 10 years or younger when they first had intercourse without stating the context that 65.9% of respondents in CYHS *never* had sexual intercourse. Furthermore, that headline figure included boys, in whom sexual initiation in the Caribbean has been consistently reported as occurring earlier than in girls [5,20,54-56]. Other regional studies have reported age at first sex as a mean, thereby failing to recognise the often skewed nature of ‘time to event’ data and possibly also failing to censor [37,57]. Our survey was in line with other Caribbean surveys reporting the same median age indicator [5,20]. The age of sexual initiation therefore remains a critical area for research influencing women’s health in the region, since age at first sex is a modifiable risk factor linked not only to IG-sex, as reported here, but also a cluster of high risk health behaviors in Caribbean girls, including smoking, alcohol and drug use, truancy, and gang membership [58-62].

Regular participation in religious worship (at least weekly) was statistically significantly associated with a lower chance of reporting a sexual encounter with any-age partner. This is consistent with previous reports in the Caribbean [63-66]. The logistic regression analysis showing a statistically significant downward trend in peer pressure index with religious service attendance, and frequency of attendance, supported the notion of an external influence of religion. However, we could not confirm an association of religious worship with IG sex. Other social network information on schools, families, influential individuals, neighbourhoods and activity groups coupled with a more comprehensive network analysis will therefore be required to better understand the impact of belief systems on the likelihood of engaging in sex or inter-generational sex in adolescent females [67,68].

Finally, it was noted that a cluster of psycho-social variables - self-esteem, sense of personal power, and peer pressure – were differentially associated with IG-sex *vs.* sex with any-age partner. The three variables were related to each other and to sex with any-age partner, yet the cluster fractured when applied to IG-sex, with low self-esteem solely associated with IG-sex. The survey also identified a strong association of multiple partnering with IG-sex. It should be noted, however, that the direction of the associations with multiple partners and self-esteem were not clear: was low self-esteem or multi-partnering a predictor of IG-sex? Or was exposure to IG-sex a cause of low self-esteem or multiple partnering?

We acknowledge limitations of this relatively small cross sectional study. The directionality of association cannot be inferred from this cross sectional survey. For example, low self-esteem might be a reason for early sexual experience or for involvement in IG sex. On the other hand, early sexual experience and involvement in IG sex might be a reason for having low self-esteem. Further qualitative research will be required to tease out causality and motivating factors and how these might differ between intergenerational relationships and those with any-age partner. We acknowledge that the dividing line can be blurred as to what constitutes transactional sex and what constitutes the normal exchange of gifts in a loving relationship. Hence, we caution against drawing the conclusion that the receipt of money or gifts was necessarily a risk factor for IG-sex, at least based on this survey. We acknowledge that the use of an interviewer may contribute a source of bias in our survey, although we sought to mitigate against this possibility by using experienced youth commissioners and training interviewers not to lead participants in their responses.

## Conclusion

In conclusion, the most powerful predictor of IG-sex in this survey was age at first sex, with the risk being halved for each additional year that sexual initiation was delayed. Although not a widely reported indicator, a proportion of 13.2% of girls having IG-sex at sexual initiation was undoubtedly high. Together with evidence here and across the Caribbean of widespread sexual coercion at first sex, with concominantly diminished condom use, this brings into sharp focus what protective programmes can be devised, besides risk reduction strategies, to help Caribbean girls re-balance the power in relationships particularly during their first sexual encounter [4,5,49]. The promotion of self-esteem in young women has been highlighted as a positive prevention approach in recently formulated policy by the National HIV/AIDS Commission of Barbados designed to remove gender stereotypes and boost self-acclamation of young women within more equitable sexual relationships [69].

## Competing interests

None of the authors disclose any competing interests from any organization, investments or patents that may in any way gain or lose financially from the publication of this manuscript. RCL discloses that he is president of the The Caribbean Cytometry & Analytical Society, a registered Barbadian HIV charity (Barbados charitable organization # 660), that funded this study and paid the article-processing fees of this manuscript. None of the authors declare any non-financial competing interests (political, personal, religious, ideological, academic, intellectual, commercial or any other) in relation to this manuscript.

## Authors’ contributions

ND made substantial contributions to the study design, acquisition and analysis of data, as well as intellectual content. CP contributed to conception, design, ethical approval, analysis of data and intellectual content. AK contributed to conception, design and interpretation of data, and intellectual content. KQ contributed to ethical approval, analysis, interpretation of data and critical revision of the manuscript. CC oversaw data collection, quality control and data management for the study. RP contributed to the conception, design and intellectual content of the study. IRH contributed to the design and led the statistical analysis of the study, as well as critical revision and intellectual content. RCL drafted the manuscript and contributed to the conception, design, and interpretation of the study, analysis of the data, and intellectual content of the manuscript. All authors read and approved the final manuscript.

## Pre-publication history

The pre-publication history for this paper can be accessed here:

http://www.biomedcentral.com/1472-6874/13/53/prepub
